# Efficacy of autologous platelet-rich plasma in treating patients with burn wounds: A protocol for systematic review and meta-analysis

**DOI:** 10.1097/MD.0000000000025650

**Published:** 2021-04-30

**Authors:** Yan-Hong Wu, Li-Ming Zhang, Yu-Zhi Wang, Jian-Wu Chen, Bin Zhang, Jian-Bing Tang, Biao Cheng

**Affiliations:** Department of Burn and Plastic Surgery, General Hospital of Southern Theater Command, PLA, Guangzhou, Guangdong Province, China.

**Keywords:** autologous platelet-rich plasma, burns, systematic review, wound healing

## Abstract

**Background:**

Burns are still regarded among severe health problems related to high morbidity and mortality rates globally. In essence, health problems associated with burns can cause significant economic burden to society. Regardless of treatment available options, no best treatment was considered adequate for treating severe burns. In particular, only a few studies have focused on the effect of autologous platelet-rich plasma to treat burn wounds. The present study aim to systematically review existing literature to examine the effectiveness and safety of autologous platelet-rich plasma (PRP) to treat burn wounds.

**Methods:**

For this study, we will conduct a systematic search using MEDLINE, EMBASE, the Cochrane Library, Web of Science, CINAHL, as well as Scopus to discover randomised controlled trials (RCTs) for the examination of effectiveness and safety of autologous PRP to treat burn wounds from their inception to March 2021 with no language restrictions. Additionally, we will search Google Scholar, ClinicalTrials.gov, as well as the reference lists of studies considered in the research to ascertain possibly eligible studies. We used two independent authors to evaluate studies for inclusion and conduct data extraction. We intend to assess study bias and quality utilizing the Cochrane Collaboration's Risk of Bias Tool 2.0. Also, we will pool study results using the fixed-effects model or random-effects model. Finally, any disagreements emanating from the process will be addressed through discussion or using a third author to mediate situations leading to disagreement.

**Results:**

The study aims at assessing the effectiveness and safety of autologous PRP for treating burn wounds.

**Conclusion:**

The study will provide specific substantiation to assess autologous PRP's effectiveness and safety in treating patients with burn wounds.

**Ethics and dissemination:**

The study does not require ethical approval since no published studies are used in it.

**OSF registration number:**

March 29, 2021.osf.io/74z5u. (https://osf.io/74z5u/)

## 1 Introduction

Burns are among the severe public health problems, with significantly higher morbidity and mortality levels.^[1–3]^ Burn wounds resulting from burns can be traumatic and challenging to manage, primarily owing to the complications arising from the initial skin loss, including pain and itching.^[4]^ Besides, hypertrophic scars induced by burns can cause physical and psychological trauma to patients, reducing their self-esteem and affecting their quality of life.^[5]^ In particular, the treatment of burns consumes large amounts of medical resources, causing a significant economic burden to society.^[2,6]^ following effectual and appropriate treatment, several patients can enjoy a quality life. To this end, burn treatment aims to ascertain effectual wound management, which mainly establishes patients’ wound survival and prognosis after experiencing severe burns.^[7,8]^ Different healing drugs, such as DNA, siRNA, growth factors, and stem cell therapy, are considered to stimulate burn wound restoration.^[9–13]^ Finding a suitable dressing for the burn wound is a significant challenge. While achieving the primary goal of wound healing, reducing the cost of burn treatment is also a critical goal to consider.

Platelet-rich plasma (PRP) denotes a plasma fraction of autologous blood with an above-the-baseline platelet concentration.^[14]^ PRP has been primarily applied in spine, plastic surgery, diabetic foot ulcers, as well as wound care. Accordingly, its use has not been associated with adverse reactions.^[15–17]^ Recent advances in PRP in humans have indicated that PRP in burns is an area of research interest. However, there are many controversies regarding the application of autologous PRP. The present study's overall aim will be to summarise the available proofs in assessing the efficacy and safety of autologous PRP to treat patients who have burn wounds.

## 2 Methods

According to the Preferred Reporting Items for Systematic Review and Meta-Analyses Protocols (PRISMA-P) statement guidelines, the protocol will be reported in the present study.^[18]^ Moreover, this protocol has been registered on the Open Science Framework (OSF, http://osf.io/).

### 2.1 Eligibility criteria

#### 2.1.1 Types of studies

Only randomised controlled trials (RCTs) assessing autologous PRP's efficacy and safety to treat patients with burn wounds will be included.

#### 2.1.2 Types of participants

Participants diagnosed with burn, including different sites and depths of burn.

#### 2.1.3 Types of interventions and comparisons

We intend to include RCTs comparing local injections or dressed with autologous PRP with no intervention, silver sulfadiazine, saline, placebo dressings, or other wound dressings.

#### 2.1.4 Types of outcome measures

The significant outcomes will include the high number of patients whose wounds heal totally. The minor outcomes were time to complete wound healing, reactions related to allergies, loss of graft, experiencing wound pain, quality or nature of scar, and adverse events.

### 2.2 Search methods for primary studies

#### 2.2.1 Electronic searches

We will conduct a systematic search using MEDLINE, EMBASE, the Cochrane Library, Web of Science, CINAHL, and Scopus to detect randomised controlled trials (RCTs) that the assessment of effectiveness and safety of autologous PRP in treatment of burn wounds from their inception to March 2021 with no language restrictions. The study will implement these search phrases: “autologous platelet-rich plasma” OR “platelet-rich plasma” OR “platelet-rich” OR “platelet plasma” OR “platelet gel” paired with “burns” OR “burn.”

#### 2.2.2 Searching other sources

Also, we will search Google Scholar, ClinicalTrials.gov, with the identity possibly eligible.

### 2.3 Data collection and analysis

#### 2.3.1 Selection of studies

**Figure 1:**
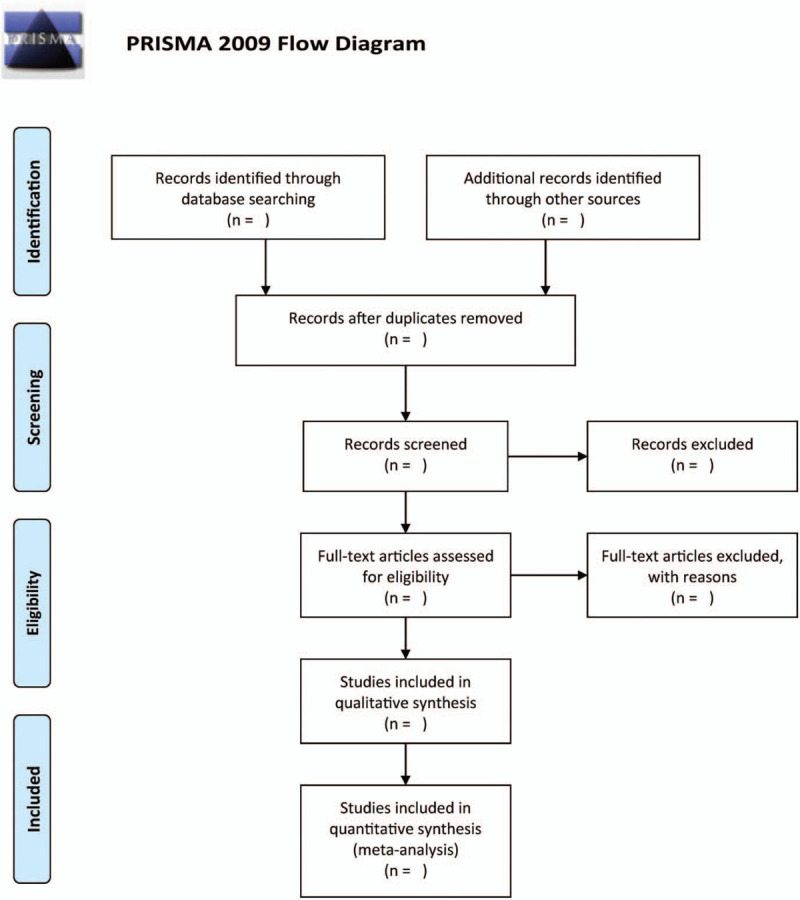
Flow diagram of study selection.

Two independent reviewers will be employed to screen the literature. They will delete duplicated and non-RCTs studies by screening titles and abstracts of the retrieved articles for suitability for RCTs. Second, the reviewers will be tasked with assessing the full texts to obtain eligible papers. Finally, we plan to address any disagreements emanating from this process through discussion. Moreover, a third author will mediate situations of misunderstandings. The selection process will be shown in Figure 1.

#### 2.3.2 Data extraction

We will use two independent reviewers to extract data from specifically included studies by means of a data extraction sheet. We will consider extracted information to contain publication details, study eligibility criteria, study details, participant characteristics, description of intervention and comparison, and outcome indicators. Moreover, we intend to address or any disagreements through discussion. A third author will mediate situations of disagreement.

#### 2.3.3 Risk of bias assessment

We will use two independent reviewers to evaluate the risk of bias in the RCTs utilizing the Cochrane Collaboration's Risk of Bias Tool 2.0. In case of any disagreements, we will address them through discussion. A third author will mediate situations of disagreement. The reviewers will review and score each of the records as ‘high’, ‘low’, or ‘unclear’ risks of bias depending on the following domains: ‘random sequence generation and allocation concealment’ (selection bias), ‘participant and personnel blinding’ (performance bias), ‘incomplete outcome data’ (attrition bias), ‘blinding for outcome assessments’ (detection bias), ‘selective outcome reporting’ (reporting bias), among others.

#### 2.3.4 Measures of treatment effect

The study will express the dichotomous data as the ‘relative risk’ (RR) and 95% ‘confidence intervals’ (CIs). It will also express continuous data as the ‘mean difference’ (MD) or ‘standardised mean difference’ (SMD) together with 95% CI.

#### 2.3.5 Management of missing data

In scenarios where of missing data, we intend to communicate with the corresponding author to obtain such missing data. Failure to recover sufficient data can impel us to examine studies with missing data and report the reasons for such scenarios.

#### 2.3.6 Assessment of heterogeneity

We plan to evaluate statistical heterogeneity by the I^2^ statistic. Furthermore, we plan to regard a level of heterogeneity of more than 50% as substantial heterogeneity. We will pool data by means of the random-effects model.

#### 2.3.7 Sensitivity analysis

By identifying sufficient studies, we intend to carry out sensitivity analysis using appropriate methods to evaluate the results’ reliability.

## 3 Discussion

Over the years, utilization of PRP has attracted widespread use in patients with burn wounds. However, the efficacy and safety of autologous PRP in treating patients with burn wounds are still inconclusive. Therefore, the present study seeks to evaluate autologous PRP's effectiveness and safety in burn wounds patients. We anticipate that these findings will provide clinicians with the basis for autologous PRP of burn wounds and provide optimal patient treatment choice.

## Author contributions

**Conceptualization:** Yan-Hong Wu, Yu-Zhi Wang.

**Data curation:** Yan-Hong Wu, Li-Ming Zhang, Yu-Zhi Wang.

**Formal analysis:** Yan-Hong Wu, Jian-Wu Chen, Biao Cheng.

**Funding acquisition:** Yan-Hong Wu, Li-Ming Zhang, Jian-Wu Chen, Bin Zhang, Jian-Bing Tang.

**Investigation:** Yan-Hong Wu, Bin Zhang.

**Methodology:** Yu-Zhi Wang, Bin Zhang.

**Project administration:** Yu-Zhi Wang.

**Resources:** Li-Ming Zhang, Jian-Wu Chen, Bin Zhang, Jian-Bing Tang, Biao Cheng.

**Software:** Li-Ming Zhang, Yu-Zhi Wang.

**Validation:** Yan-Hong Wu, Li-Ming Zhang, Jian-Wu Chen, Bin Zhang, Jian-Bing Tang, Biao Cheng.

**Visualization:** Yu-Zhi Wang, Jian-Wu Chen, Biao Cheng.

**Writing – original draft:** Yan-Hong Wu.

**Writing – review & editing:** Yan-Hong Wu.
